# Negative consequences of sleep disturbance in individuals with limb loss

**DOI:** 10.1007/s11325-026-03856-z

**Published:** 2026-11-11

**Authors:** Marisa Pontillo, Carla Kinnunen, Michelle D. Lockwood, Shawn Farrokhi, Sara R. Gorczynski, Courtney M. Butowicz

**Affiliations:** 1 Naval Medical Center San Diego, 34500 Bob Wilson Drive, San Diego, CA USA; 2 Extremity Trauma and Amputation Center of Excellence, Defense Health Agency, Falls Church, VA USA; 3 Department of Physical Therapy, University of Southern California, Los Angeles, CA USA; 4 Center for the Intrepid, San Antonio, TX USA; 5 Department of Physical Therapy, Chapman University, Irvine, CA USA; 6 Henry Jackson Foundation for the Advancement of Military Medicine, Inc, Bethesda, MD USA; 7 Walter Reed National Military Medical Center, Bethesda, MD USA

**Keywords:** Sleep, Sleep disturbance, Psychological outcomes, Military medicine, Amputations

## Abstract

**Objectives:**

To identify the association between sleep disturbance with physical function and psychological outcomes in individuals with limb loss.

**Methods:**

This cross-sectional study included 218 community ambulating individuals with at least one amputation, recruited from Military treatment facilities, community events, and prosthetic clinics. Participants’ demographics were obtained from self-reported questionnaires. The Patient-Reported Outcomes Measurement Information System (PROMIS)-29 scores were used to assess sleep disturbance, physical function, anxiety, depression, fatigue, pain interference, and social participation. Sleep disturbance data were used to categorize the participants into three mutually exclusive groups: no sleep disturbance, mild sleep disturbance, and moderate to severe sleep disturbance. One-way analyses of variance (ANOVA) were used to determine if differences existed in PROMIS scores between the three sleep disturbance categories.

**Results:**

Results showed that as compared to individuals with no sleep disturbance, those with moderate to severe sleep disturbance had worse physical function; higher levels of anxiety, depression, fatigue, and pain interference, and less social participation. In addition, a difference was noted between the mild and the moderate to severe groups indicating worse depression, fatigue, social participation, and pain interference. When compared to the no sleep disturbance group, the mild disturbance group demonstrated worse physical function and fatigue (all *P* < 0.05).

**Conclusions:**

Results reflect that higher levels of sleep disturbance are associated with worse functional and psychological outcome measures in individuals with limb loss. Findings from this study also indicate that a higher threshold of sleep disturbance is required to impact levels of depression, social participation, and pain interference.

## Introduction

Sleep is an essential part of health and well-being such that poor sleep quality may negatively impact nearly all body systems and functions [1–8]. For example, poor sleep quality and duration is linked to hypertension, heart disease, and stroke [9]. Sleep also affects cognitive function as quality sleep improves attention, decision-making, and learning [10]. Additionally, poor sleep quality can impair cognitive functioning, increase pain perception [5, 6], and lead to greater psychosocial distress and catastrophizing [11]. Insufficient sleep is both a risk factor and symptom of conditions like depression, anxiety, and post-traumatic stress syndrome, further illustrating the negative impacts sleep impairments may have on psychological health [12]. These, in aggregate, demonstrate that sleep quality and duration are critical health outcomes when assessing an individual’s quality of life, especially considering chronic short sleep duration is associated with higher all-cause mortality [1].

Sleep disturbances are prevalent across clinical populations including individuals with neurological diseases (e.g., multiple sclerosis) and chronic pain conditions and have been associated with a range of negative health outcomes, including contributing to increased neuroinflammation that may accelerate disease progression [13]. Associations between sleep and pain have been studied more extensively; pain impairs sleep continuity, and insufficient or fragmented sleep amplifies pain sensitivity and lowers pain thresholds [6]. There is a significant, positive association between sleep disturbance and pain in various populations including those with chronic pain or lower limb loss [3, 7, 14, 15]. Current literature endorses a bidirectional link between sleep and pain modulated by neural and physiological mechanisms including elevated levels of proinflammatory cytokines (e.g., interleukin-6, tumor necrosis factor-alpha) and dysregulation of neurotransmitters such as serotonin, dopamine, and gamma-aminobutyric acid, which are integral to both sleep regulation and nociception [4, 6, 7, 16, 17, 7, 16]. These changes are associated with heightened inflammation, cognitive decline, depression, and increased vulnerability to pain [18]. Among persons with limb loss, chronic residual limb pain is associated with inflammatory biomarkers such that increased pain intensity is positively correlated with proinflammatory mediators (i.e., interleukin-8, tumor necrosis factor-alpha​) while the relationship between pain catastrophizing and pain intensity are partially mediated by proinflammatory markers, suggesting inflammatory mechanisms or responses may be, in part, underlying pain mechanisms [19]. Together, these findings underscore the complex, reciprocal interplay between sleep and health outcomes (physiological and psychological) in clinical populations, highlighting the need for integrated rehabilitation strategies that target both sleep and pain mechanisms [5, 6, 13, 15, 19].

Sleep quality is often not considered as a critical factor in rehabilitation after limb loss even though existing research on those with limb loss shows poor sleep quality and increased risk of developing insomnia and chronic pain compared to control groups [16]. Rather, rehabilitation for individuals with limb loss typically concentrates on physical impairments, mobility deficits, and activity limitations [14], ​despite that over two million people currently live with limb loss in the United States [20]. Importantly, higher sleep disturbance scores have been associated with higher anxiety among individuals with lower limb loss, a population that is 31.7% more likely to report poor sleep quality compared to healthy controls [9].

Although previous studies have examined the association between sleep quality and quality of life in various populations, the negative consequences of sleep disturbance have not been extensively evaluated in individuals with limb loss [4]. This study investigates the interaction of biopsychosocial factors such as physical function, anxiety, depression, fatigue, pain interference, and social participation that contribute to sleep disturbance in individuals with limb loss. The authors hypothesize that sleep disturbance is associated with poorer physical function, mental health, and overall quality of life in this population. By demonstrating these links, our findings aim to highlight the critical importance of addressing sleep disturbance in rehabilitation strategies, as its adverse effects may significantly undermine the effectiveness of patient care.

## Methods

This cross-sectional study consisted of 218 community ambulating individuals who participated in a larger, 8-year study (2017–2024) assessing the differences in function and wellness of individuals with amputations treated within civilian and military healthcare systems. Participants were screened for eligibility and were included if they had at least one major upper or lower limb amputation, could speak and read English, and were at least 18 years of age at the time of data collection. Individuals with only digit amputations [finger(s) or toe(s)] were excluded. Eligible participants provided written consent approved by the Naval Medical Center San Diego Institutional Review Board (NMCSD.2017.0039).

Participants were recruited by convenience sampling from Military treatment facilities, community events, and local prosthetic clinics. The three Military treatment facilities included Naval Medical Center San Diego, Walter Reed National Military Medical Center, and Center for the Intrepid/Brooke Army Medical Center. Community events included the Amputee Coalition National Assembly, events with the Challenged Athletes Foundation, Texas Regional Games, and the American Orthotic and Prosthetic Association National Assembly.

All data for this study were collected via questionnaires face-to-face at the aforementioned locations and events. Demographic factors included age, sex, height, weight, body mass index (BMI; calculated), and setting where the individual received rehabilitation care (Military or civilian healthcare facility). Limb loss characteristics included time since amputation and level of amputation (bilateral lower / unilateral lower/ bilateral upper/ unilateral upper/ multiple).

The Patient-Reported Outcomes Measurement Information System (PROMIS)-29 Profile v1.0 short form T-scores were used to assess sleep disturbance, physical function, anxiety, depression, fatigue, pain interference, and social participation. PROMIS is intended for clinicians and researchers in various disciplines to provide reliable, valid, and flexible measures of physical, mental, and social health among individuals with various chronic conditions [21, 22]. All PROMIS scores are reported on a T-score metric with a T-score of 50 aligned with the general population mean and a standard deviation of 10. Higher scores indicate more of the quantity being assessed. The PROMIS has shown to have excellent reliability and moderate to high responsiveness and the minimal clinically important difference for PROMIS domains ranges from 1.9 to 3.4. Additionally, the PROMIS Sleep Disturbance measure has demonstrated excellent reliability and validity across clinical populations, with high internal consistency and strong test-retest reliability [23, 24]. T-scores for the sleep disturbance short form were used as previously described to categorize the participants into the following three mutually exclusive groups: no sleep disturbance (< 55), mild sleep disturbance (55-59.9), and moderate to severe sleep disturbance (60+) [6].

### Statistical analysis

The present study was a secondary analysis of prospectively collected data from the larger study; therefore, an a priori sample size calculation specific to the present analysis was not performed. Demographic and limb loss characteristic data was reported by mean ± standard deviation for continuous data and as counts and frequencies for categorical data. Normality was assessed by skewness and kurtosis (between − 1 and 1); non-significant Kolmogorov-Smirnov tests; box plots and normal Q-Q plots. One-way analyses of variance (ANOVA) were used to determine if differences existed in physical function, anxiety, depression, fatigue, pain interference, and social participation between the three self-reported sleep disturbance categories. Tukey’s post hoc test was used for pairwise comparisons. Effect sizes were calculated to determine the magnitude of relationships (Cohen’s *d*), interpreted as 0.2 = small, 0.5 = medium, 0.8 = large. Significance was set to a priori to α = 0.05.

## Results

**Fig. 1:**
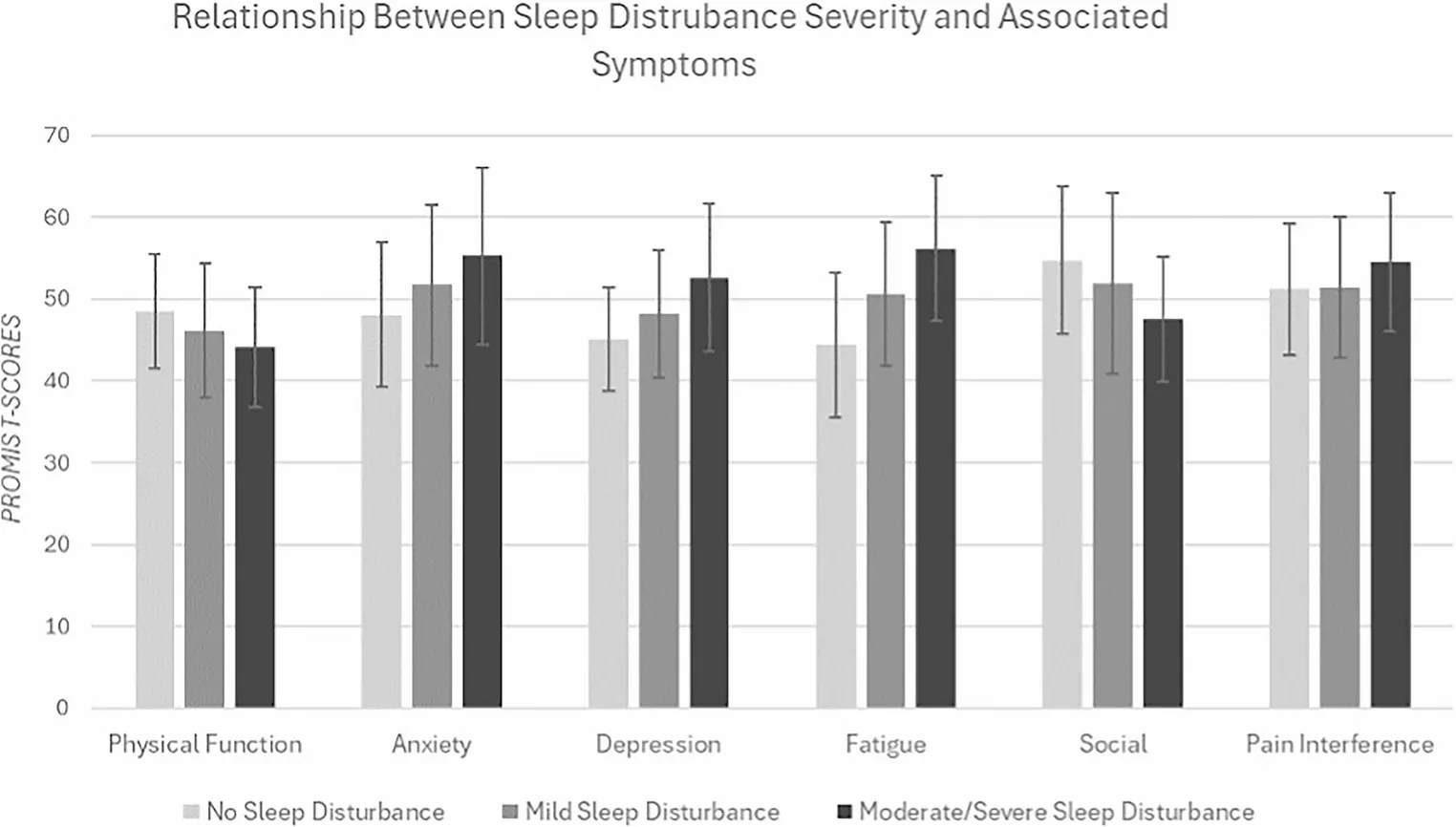
PROMIS subscale scores, categorized by sleep disturbance category

Demographics and characteristics are included in Table 1; patient reported outcomes by sleep category and group differences are reported in Table 2. Overall, worsening sleep disturbance demonstrated a graded association with poorer PROMIS outcomes across physical, psychological, and social domains. Compared with participants with limb loss categorized as having no sleep disturbance, those with moderate to severe sleep disturbance had worse physical function; higher levels of anxiety, depression, fatigue, and pain interference, and less social participation, (all *P* < 0.05). In addition, a difference was noted between the mild and the moderate to severe groups indicating worse depression, fatigue, social participation, and pain interference (all *P* < 0.05). When compared to the no disturbance group, the mild sleep disturbance group demonstrated worse physical function and fatigue (all *P* < 0.05), but differences were not seen between other PROMIS domains (Fig. 1).

**Table 1:** Demographics by categorized level of sleep disturbance

	Level of Sleep Disturbance		
	No Sleep Disturbance	Mild Sleep Disturbance	Moderate to Severe Sleep Disturbance
N	159	36	23
Age (years)	38.6 (12.9)	37.3 (9.7)	40.1 (13.3)
Sex (males/females/not reported)	113 / 46	28 / 7 / 1	13 / 10 / 0
BMI *(kg/m*^*2*^*)*	26.5 (5.9)	26.7 (4.6)	30.3 (7.5)
Amputation Type (bilateral lower / unilateral lower/ bilateral upper/ unilateral upper/ multiple)	15 / 111 / 2 / 22 / 9 *9.4 / 69.8 / 1.3 / 13.8 / 5.7%*	7 / 24 / 1 / 3 / 1 *19.4 / 66.7 / 2.8 / 8.3 / 2.8%*	0 / 16 / 1 / 4 / 2 *0 / 69.6 / 4.3 / 17.4 / 8.7%*
Time to Amputation (months)	143.8 (158.0)	97.6 (109.8)	126.7 (147.0)
Rehabilitation Setting (military Service members/ civilian/ did not attend/ not reported)	43 / 94 / 21 / 1 *27.0 / 59.1 / 13.2 / 0.6%*	21 / 13 / 1 / 1 *58.3. / 36.1 / 2.8 / 2.8%*	9 / 12 / 2 / 0 *39.1 / 52.2 / 8.7 / 0%*
PROMIS Sleep Interference Score	45.9 (7.1)	57.4 (1.4)	67.0 (4.5)

*Continuous data reported in mean (standard deviation) and categorical data as counts (frequencies in italics). Abbreviations: BMI = body mass index, kg = kilograms, m = meters*

**Table 2:** Patient reported outcomes by sleep category and group differences

PROMIS Subscale and ANOVA Results	Comparison	Pairwise Post-Hoc Comparisons (P)
PROMIS Physical Function F = 5.83, *P* < 0.01, *d=*-0.05	*no sleep disturbance* to *mild sleep disturbance*	0.03*
	*no sleep disturbance* to *moderate to severe sleep disturbance*	< 0.01*
	*mild sleep disturbance* to *moderate to severe sleep disturbance*	0.37
PROMIS Anxiety F = 6.36, *P* < 0.01, *d =* 0.06	*no sleep disturbance* to *mild sleep disturbance*	0.13
	*no sleep disturbance* to *moderate to severe sleep disturbance*	< 0.01*
	*mild sleep disturbance* to *moderate to severe sleep disturbance*	0.07
PROMIS Depression F = 11.33, *P* < 0.01, *d=-*0.10	*no sleep disturbance* to *mild sleep disturbance*	0.08
	*no sleep disturbance* to *moderate to severe sleep disturbance*	< 0.01*
	*mild sleep disturbance* to *moderate to severe sleep disturbance*	0.01*
PROMIS Fatigue F = 21.50, *P* < 0.01, *d=*-0.17	*no sleep disturbance* to *mild sleep disturbance*	< 0.01*
	*no sleep disturbance* to *moderate to severe sleep disturbance*	< 0.01*
	*mild sleep disturbance* to *moderate to severe sleep disturbance*	0.02*
PROMIS Social F = 9.02, *P* < 0.01, *d =* 0.08	*no sleep disturbance* to *mild sleep disturbance*	0.09
	*no sleep disturbance* to *moderate to severe sleep disturbance*	< 0.01*
	*mild sleep disturbance* to *moderate to severe sleep disturbance*	0.02*
PROMIS Pain Interference F = 4.73, *P* = 0.01, *d=*-0.04	*no sleep disturbance* to *mild sleep disturbance*	0.63
	*no sleep disturbance* to *moderate to severe sleep disturbance*	< 0.01*
	*mild sleep disturbance* to *moderate to severe sleep disturbance*	0.03*

*Indicates statistically significant at P < 0.05. Bold indicates the group with the poorer score*

Effect sizes across outcomes were small. Fatigue exhibited the largest between-group effect (d = − 0.17), followed by depression (d = − 0.10) and social participation (d = 0.08), suggesting that fatigue demonstrated the greatest magnitude of association with sleep-disturbance category.

## Discussion

Sleep disturbance is associated with adverse physical and psychological health outcomes, including increased pain, fatigue, and psychological distress, which may be particularly consequential for individuals with limb loss who already experience substantial physical and psychosocial demands. However, the relationship between sleep disturbance and multiple domains of health has not been extensively evaluated in this population. Therefore, this study sought to investigate the associations between self-reported sleep disturbance and physical function, anxiety, depression, fatigue, pain interference, and social participation in individuals with limb loss. By examining these domains concurrently, this study provides a more comprehensive understanding of the relationship between sleep disturbance and biopsychosocial health following limb loss.

Consistent with findings in broader populations with chronic illness or pain, our results demonstrate that individuals with limb loss and moderate to severe sleep disturbance report poorer scores on all PROMIS subscales: worse physical function; higher levels of anxiety, depression and fatigue; increased pain interference and reduced social participation. These associations reinforce previous literature suggesting that sleep quality plays a pivotal role in overall health and well-being, and they extend these findings to individuals with limb loss—a group in which the effects of sleep disturbance have been understudied. This study offers important novel contributions by focusing on the often-overlooked relationship between sleep disturbance and health outcomes specific to the limb loss population. While prior research has linked poor sleep to adverse outcomes in chronic pain [3, 4, 6, 7, 16] multiple sclerosis [5], or aging populations [25], few studies have systematically examined these connections in individuals with limb loss. This is despite evidence that people with limb loss face elevated risks for poor sleep, pain, and reduced function [15].

The PROMIS questionnaire [26] provides a way to measure and quantify sleep disturbance​ and the interconnectedness of physical and mental health factors contributing to pain and quality of life with sleep disturbance. By employing PROMIS to evaluate a range of health domains, our study provides a broad, integrated view of how sleep disturbance relates to biopsychosocial outcomes in this population. Importantly, our findings highlight a potential threshold effect: moderate to severe sleep disturbance was linked to pronounced declines in depression, pain interference, and social participation. Notably, fatigue and physical function were sensitive even to mild sleep disturbance, suggesting that early sleep problems may impact energy and mobility, which are critical to functional independence and quality of life after limb loss. These nuanced observations suggest that sleep disturbance may meaningfully contribute to poorer rehabilitation outcomes, yet it is not routinely assessed or targeted in limb loss rehabilitation programs. This gap in standard care presents a significant opportunity to enhance rehabilitation strategies by incorporating sleep-focused assessments and interventions.

The lack of differences between some of the adjacent groups may be explained by several factors: threshold effects, overlapping symptom severity, and the sensitivity of individual PROMIS domains. Certain outcomes, such as fatigue and physical function, appear to be more sensitive to even mild sleep disturbance, as evidenced by significant differences between the no sleep disturbance and mild sleep disturbance groups. In contrast, domains such as depression, anxiety, pain interference, and social participation may require a greater degree or longer duration of sleep impairment before clinically meaningful differences emerge, which may explain why significant differences were primarily observed between the no disturbance and moderate-to-severe disturbance groups. PROMIS subscales are reported as T-scores and thus reflect the sample score distribution [25]. For physical function, both the no and mild sleep disturbance groups have mean scores which fall within normal limits, while the moderate to severe group’s scores indicate a mild level of disability. The same holds true for the anxiety and fatigue subscales. Interestingly, all three groups report scores which fall within normal limits for social participation, depression and pain interference, and thus the PROMIS T-score structure and the relatively high-functioning nature of this cohort may have contributed to the absence of differences in some comparisons. However, it should be noted that PROMIS T scores are for the *sample* and not the population and were established from individuals with chronic conditions. As this study was part of a larger study which investigated community ambulating individuals with limb loss, it is not unexpected that this cohort reported better physical and psychosocial function. Regardless, the differences seen between levels of sleep disturbance capture that these facets are interconnected.

Given the chronic nature of limb loss rehabilitation, the finding that participants were, on average, more than 11 years post-amputation, highlights that sleep disturbance is not limited to the immediate post-operative or early rehabilitation period. Rather, sleep-related impairments may persist for years and continue to negatively influence physical function, pain, fatigue, and psychosocial health long after initial recovery. These findings support the need for routine, long-term sleep screening throughout the continuum of care for individuals with limb loss, rather than limiting assessment to the acute phase following amputation. Incorporating ongoing sleep evaluation into long-term follow-up and rehabilitation programs may help clinicians identify persistent deficits earlier and implement interventions that improve long-term health and quality of life.

These findings carry important clinical implications. Despite the known importance of sleep for recovery and rehabilitation, sleep quality is not routinely assessed or targeted in limb loss rehabilitation programs. Our findings suggest that integrating sleep assessments and interventions into the standard care of individuals with limb loss may offer a pathway to improving outcomes across physical, emotional, and social domains. Interestingly, the mean time since amputation was just over 11 years for the study cohort, indicating that sleep disturbance and other deficits persist long after onset. Addressing sleep as a modifiable factor—through behavioral interventions, environmental modifications, and patient education—could enhance rehabilitation effectiveness and long-term well-being.

This study has several limitations. First, the cross-sectional design limits the ability to determine causal or directional relationships between sleep disturbance and the physical and psychosocial outcomes assessed. Second, all data were collected via validated patient-reported measure rather than objective sleep measures such as actigraphy or polysomnography,, which are subject to recall bias, reporting inaccuracies, and potential influence of participant perceptions and cannot characterize objective sleep duration, architecture, or sleep-wake patterns. Third, although PROMIS measures are validated and widely used, the interpretation of T-scores is relative to reference populations, which may limit direct comparison with normative or clinical groups outside the present cohort. Fourth, the sample consisted of community-ambulating individuals with limb loss recruited primarily through military treatment facilities, community events, and prosthetic clinics. As such, the cohort may represent individuals who are more engaged with rehabilitation services and may not reflect the broader limb loss population, including those with more severe disability, limited mobility, or reduced access to care. Fifth, heterogeneity in amputation level, time since amputation, and rehabilitation setting may have introduced unmeasured variability that could influence the associations observed. Finally, factors not assessed in this study—such as prosthetic comfort, medication use, comorbid conditions, or environmental influences—may also contribute to sleep disturbance and its consequences.

Despite these limitations, this study provides novel evidence that sleep disturbance is linked to poorer physical and psychological health outcomes in individuals with limb loss. The findings underscore the critical need for further research into the role of sleep disturbance in the health and rehabilitation outcomes of individuals with limb loss. Future studies should prioritize longitudinal designs to explore causative relationships and the effects of targeted sleep interventions on functional and psychosocial outcomes in this population. Such research could clarify whether sleep disturbance precedes declines in function and mental health, or if it emerges because of these impairments, or both. Additionally, there is a pressing need to evaluate targeted interventions aimed at improving sleep quality among individuals with limb loss. Trials investigating the effectiveness of behavioral, pharmacological, and technology-based sleep interventions (including, but not limited to, cognitive-behavioral therapy for insomnia, prosthetic adjustments to enhance comfort) could provide valuable insight into strategies that may mitigate the broad negative effects associated with poor sleep. Further exploration of moderating and mediating factors—including pain characteristics, prosthetic fit and comfort, physical activity levels, and psychosocial supports—may illuminate why some individuals with limb loss are more vulnerable to sleep disturbance and its consequences. Lastly, other factors-both those which improve sleep duration and quality and those which are related to sleep disturbance not measured in this stud​y-may also provide further insights into this complex relationship. Ultimately, advancing this line of research could inform more holistic and personalized rehabilitation approaches, with sleep health incorporated as a key component of post-amputation care to improve long-term outcomes in physical, emotional, and social domains.

In conclusion, greater self-reported sleep disturbance was associated with poorer physical, psychological, and social health outcomes among individuals with limb loss. Moderate-to-severe sleep disturbance was associated with worse outcomes across all assessed PROMIS domains, while even mild sleep disturbance was associated with poorer physical function and greater fatigue. These findings demonstrate that sleep disturbance is associated with multiple dimensions of health and functioning in individuals with limb loss.

## Data Availability

Data will be made available on reasonable request.

## Declarations

### Copyright statement

I am a military Service member or employee of the U.S. Government. This work was prepared as part of my official duties. Title 17, U.S.C., § 105 provides that copyright protection under this title is not available for any work of the U.S. Government. Title 17, U.S.C., § 101 defines a U.S. Government work as a work prepared by a military Service member or employee of the U.S. Government as part of that person’s official duties.

### AI statement

No AI was used in the development of this manuscript.

### Conference presentation

This data was presented, in part, at American Physical Therapy Association’s Combined Sections Meeting, Boston, MA, February 2023.
